# Systematic assessment and optimizing algorithm of tumor mutational burden calculation and their implications in clinical decision-making

**DOI:** 10.3389/fonc.2022.972972

**Published:** 2022-11-08

**Authors:** Daqiang Sun, Meilin Xu, Chaohu Pan, Hongzhen Tang, Peng Wang, Dongfang Wu, Haitao Luo

**Affiliations:** ^1^ Department of Thoracic Surgery, Tianjin Chest Hospital, Affiliated Chest Hospital of Tianjin University, Tianjin, China; ^2^ Pathology Department, Tianjin Chest Hospital, Affiliated Chest Hospital of Tianjin University, Tianjin, China; ^3^ The First Affiliated Hospital, Jinan University, Guangzhou, China; ^4^ Department of Medicine, YuceBio Technology Co., Ltd, Shenzhen, China

**Keywords:** immunotherapy, tumor mutation burden, East Asian populations, method optimizing, clinical decision-making

## Abstract

Tumor mutation burden (TMB) has been validated as a biomarker to predict the response of immune checkpoint inhibitors (ICIs) treatment in various cancers. However, the effects of different sequencing platforms, cancer types, and calculation algorithms on TMB as well as its cut-off value for predicting immunotherapy efficacy in the East Asian population still need to be further investigated. In this study, the data of 4126 samples generated by targeted panel sequencing or whole-exome sequencing (WES) in different platforms and public sequencing data from 3680 samples that contained targeted panel sequencing, WES and whole-genome sequencing (WGS) were obtained. The impact of different sequencing platforms and methods on TMB calculation was assessed. No significant bias was found in TMB calculated by different platforms. However, TMB calculated from WGS was significantly lower than those calculated from targeted panel sequencing and WES. The distribution of TMB at different sequencing depths and tumor purity were analyzed. There was no significant difference in the distribution of TMB when the sequencing depth was greater than 500, the tumor purity estimated by hematoxylin-eosin (HE) staining was between 0.1-1.0 or estimated by next-generation sequencing (NGS) was greater than 0.4. In addition, the somatic-germline-zygosity (SGZ) algorithm was optimized to calculate TMB from tumor-only sequencing samples in the East Asian population. The correlation coefficient of TMB calculated with the optimized SGZ algorithm and paired normal-tumor sequencing is 0.951. Furthermore, the optimal cut-off value of TMB in East Asian lung cancer patients treated with ICIs was determined to be 7 mut/Mb instead of 10 mut/Mb through the ROC curve and Log-rank analysis in the training cohort and validated in the test cohort. Patients with TMB ≥ 7 mut/Mb had better outcomes than patients with TMB<7 mut/Mb. In conclusion, this study systematically analyzed the factors that influenced the TMB calculation and optimized the SGZ algorithm to calculate TMB from tumor-only sequencing samples in the East Asian population. More importantly, the cut-off value of TMB for predicting immunotherapy efficacy was determined to be 7 mut/Mb instead of 10 mut/Mb in East Asian lung cancer patients, which can help in clinical decision-making.

## Introduction

The advent of immune checkpoint inhibitors (ICIs), including anti-programmed cell death protein 1 (PD-1)/programmed death-ligand 1 (PD-L1) and anti-cytotoxic T lymphocyte-associated antigen-4 (CTLA4), have revolutionized cancer therapy (1–4). Several ICIs have been approved by Food and Drug Administration (FDA) in multiple tumor types (5). However, only a subset of patients achieved durable clinical responses, and some may even suffer from unique immune-related toxicities or even hyperprogression (6–10). Therefore, predictive biomarkers were urgently required to optimize the treatment of ICIs.

The expression of PD-L1 in tumor and/or tumor-infiltrating lymphocytes assessed by immunohistochemistry (IHC) is an established biomarker to predict efficacy in the treatment of ICIs across many cancer types including melanoma, non-small cell lung cancer (NSCLC) and colorectal cancer (9, 11). However, on the one hand, PD-L1 alone as a biomarker is insufficient to distinguish responders (12); on the other hand, the detection methods and thresholds for PD-L1 expression are variable (13, 14). Therefore, new biomarkers are required to improve the treatment decision-making and identify potential responders from ICIs therapy.

Tumor mutation burden (TMB), which was defined as the number of all non-synonymous somatic mutations per megabase based on the genome examined, has been reported to predict the efficacy of ICIs therapy in multiple tumor types (15–17). The more mutations, the more neoantigens are produced, which ultimately activate the stronger antitumor immune response (18). Based on the results from the phase 2 KEYNOTE-158 trial, pembrolizumab was approved by the FDA for patients with TMB ≥10 mut/Mb. Patients with a TMB ≥10 mut/Mb were defined as TMB-high and associated with better response rates (19). However, there were racial differences in TMB across multiple cancer types (20). Compared with European and American populations, the TMB is lower in East Asian populations. The cut-off of 10 mut/Mb may lead to fewer East Asian populations meeting eligibility for the treatment of ICIs. Therefore, the association of TMB cut-off in East Asian populations with ICIs treatment outcomes needs to be further investigated.

Currently, multiple platforms and sequencing methods have been used for next-generation sequencing (NGS) (21). However, the effect of different sequencing platforms and methods on TMB calculation has not been systematically evaluated. Compared with sequencing both tumor and matched normal specimens, tumor-only sequencing could reduce time and cost. In addition, many clinical tumor samples lack matching normal tissue, which requires the development of algorithms to calculate TMB for these samples. However, the current algorithms, including somatic-germline-zygosity (SGZ), were designed based on European and American populations. For the East Asian population, the corresponding algorithm is lacking (22–24). Therefore, an algorithm for TMB calculation from tumor-only sequencing samples in the East Asian population was urgently needed.

In this study, the effect of different sequencing platforms and methods on the calculation of TMB has been systematically evaluated. To calculate TMB for Asian patients with tumor-only sequencing, we optimized the SGZ algorithm and demonstrated the reliability of calculating TMB from tumor-only sequencing samples. Furthermore, the cut-off of TMB in Asian patients was determined and its efficacy in the treatment of ICIs has been investigated.

## Materials and methods

### Samples and datasets

Data for 4126 samples sequenced with different sequencing platforms and methods were obtained from a CAP-accredited laboratory (YuceBio Technology Co., Ltd, China) (**Table S1**). To investigate the racial differences in TMB value, 3680 genomic data of European and American populations were collected from cBioPortal (25–28).

Sixty-two samples with matched control were retrospectively obtained to analyze the correlation coefficient of TMB calculated from methods of tumor-only sequencing and paired normal-tumor sequencing (**Tables S2**–**S5**). To determine the cut-off of TMB for predicting immunotherapy efficacy in the East Asian population, tumor samples of sixty-six lung patients treated with ICIs between July 2019 to September 2020 were retrospectively collected as a training cohort and sequenced without normal control (**Table S6**). Furthermore, genomic and clinical data of Sixty-nine East Asian NSCLC patients subjected to ICIs treatments were obtained as a test cohort to validate the cut-off of TMB (29). Durable clinical benefit (DCB) was defined as complete response, partial response, or stable disease (SD) that lasted for ≥ 24 weeks, and non-durable benefit (NDB) was defined as SD that lasted for< 24 weeks or progressive disease.

### Next-generation sequencing (NGS) and mutation analysis

Genomic profiling was implemented on tumor tissues and matched peripheral blood samples. The GeneReadDNA FFPE kit (Qiagen) and Qiagen DNA blood mini kit (Qiagen) were used to extract DNA from tumor specimens and blood, respectively. For tumor-only sequencing, DNA from the tumor sample was extracted with a GeneReadDNA FFPE kit (Qiagen). DNA quantification was performed with the dsDNA HS Assay Kit (ThermoFisher Scientific, USA). For the platform of Illumina, sequencing libraries were built by SureSelect XT Human All Exon V6 (Agilent) for WES or a customized next-generation sequencing panel targeting exons of 1267 genes for panel sequencing, respectively. Sequencing procedures were utilized by the NextSeq 550AR platform with 150-bp paired-end reads. For the platform of MGI, sequencing libraries were built by Exome Plus Panel V1.0 (IDT, USA) for WES or a customized next-generation sequencing panel targeting exons of 1267 genes for panel sequencing, respectively. Sequencing procedures were utilized by the MGISEQ-T7 platform with 100-bp paired-end reads.

Sequencing reads with > 10% N rate and/or > 10% bases with a quality score of< 20 were filtered using SOAPnuke (Version 1.5.6) (30). Somatic single nucleotide variants and insertions and deletions (indels) were detected using VarScan (Version 2.4) (31). Next, Bcftools (1.14) was utilized to filter possible false-positive mutations with the parameter set as follow: “basicfilter = “““‘(STRLEN(REF)>50 || STRLEN(ALT)>50) || INFO/STATUS!~”Somatic”‘“““ hotspotfilter = “““‘INFO/HOTSPOT!=“.” && ((INFO/SOR!=0 && INFO/SOR<3) || INFO/VD<5 || INFO/AF<0.007 || INFO/SSF>0.05)’”““ fpdbfilter = “““‘INFO/HOTSPOT=“.” && ((INFO/FPDB!=“0” && INFO/FPDB!=“.”) || (INFO/GERMLINE!=“0” && INFO/GERMLINE!=“.”))’”““ normalfilter = “““‘INFO/HOTSPOT=“.” && ((INFO/GERMLINE)!=“.” || (FORMAT/PMEAN [0]<20)||((INFO/SOR!=0 && INFO/SOR<5) || INFO/AF<0.02 || INFO/SSF>0.01)||(INFO/AF<0.05 && FORMAT/MQ[0]<50)||(FORMAT/MQ[0]<30)||(INFO/AF<0.05 && FORMAT/QUAL[0]<30) || ((INFO/MSI>10||(INFO/MSILEN>1 && INFO/MSI>4)) && INFO/AF<0.3)||(type!=“snp” && INFO/MSI>3 && ((INFO/MSILEN=(strlen(REF)-1))||(INFO/MSILEN=(strlen(ALT[0])-1))) && INFO/AF<0.1) || (FORMAT/NM[0]>2 && FORMAT/MQ[0]<60 && INFO/AF<0.2) || (FORMAT/NM[0]>3 && (FORMAT/MQ[0]<55||FORMAT/NM[1]>3)) || (FORMAT/DP[0]<30 || FORMAT/DP[1]<30)|| INFO/VD<10 || (FORMAT/BIAS[0:0]=“2” && FORMAT/BIAS[0:1]=“1”) || (FORMAT/SBF[0]< 0.05 && FORMAT/VD[0]<50) || ((INFO/SOR!=0 && INFO/SOR<10) && FORMAT/MQ[0]<60))’ ““““ (32). Finally, SnpEff (Version 4.3) was used to functionally annotate the mutations detected in the tumor samples (33).

TMB was determined as the number of all nonsynonymous mutations and indels per megabase of the genome examined.

### Tumor purity estimation

To estimate the tumor purity by hematoxylin-eosin (HE) staining, the sample was fixed in the 10% formalin solution, embedded in paraffin. Then the 5 µm slide was stained with HE. The tumor purity is the value of tumor cells divided by all cells. To estimate the tumor purity by NGS, the sequencing reads were quality controlled using SOAPnuke (Version 1.5.6) (30), then aligned to the reference genome using BWA (v0.7.12). The tumor purity was estimated by Ascatngs (v3.1.0) (34).

### SGZ optimization and mutation analysis

Mutations from tumor-only sequencing samples were identified by the somatic-germline-zygosity (SGZ) algorithm. For each sample, massively parallel sequencing (MPS) variant analysis was executed to create a genome-wide copy number profile, which is segmented and modeled to estimate the ploidy (Ψ) and overall tumor purity (p), as well as per segment copy number (C) and minor allele count (M). The log-ratio of variants was defined by the following formula:


$$Logratio=log_{2}\left(\frac{P*C+2*(1-P)}{P*\Psi +2*(1-P)}\right)$$

For each variant, the error log ratio was obtained by calculating the absolute value of the difference in log ratio between variant and segments. Finally, the germline variant or somatic variant was identified mainly by frequency, purity and error log ratio. However, the cut-off value of the above parameter was fit to European and American populations, which resulted in a high false-positive rate in the East Asian population (23). In order to calculate mutation from tumor-only sequencing in the East Asian population, the SGZ algorithm was optimized as followed: (1) generating a mutation background library based on the East Asian population, (2) analyzing mutations with SGZ, (3) filtering out variants that appear more than 5 times in background library, while variants with a frequency higher than 0.9 were retained.

### Statistical methods

All statistical analyses were implemented in Python (3.10.1). An independent *t*-test was used to compare TMB values between different groups. Correlation analysis was performed using the Pearson correlation analysis. Roc-curve and Log-rank test analyses were conducted to determine the cutoff of TMB. Categorical variables were evaluated with the Fisher-exact test. Kaplan-Meier curve, Log-rank test, and Cox regression were used to determine the significance of TMB on overall survival (OS) and Progression-Free-Survival (PFS). Statistical significance was set at p-value< 0.05.

## Results

### Effects of different sequencing platforms, sequencing methods and races on TMB values

**Figure 1:**
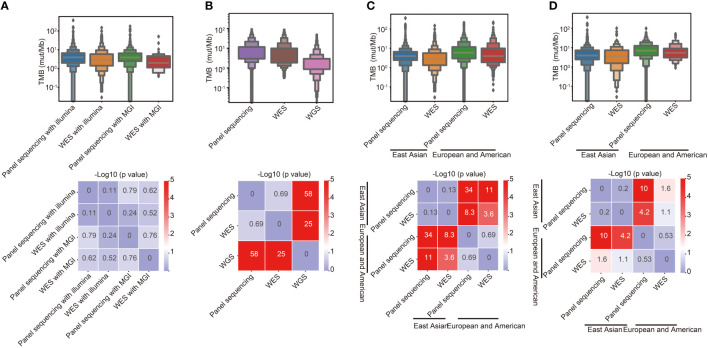
Effects of different sequencing platforms, sequencing methods and races on TMB. **(A)** Comparison of TMB between different sequencing platforms and methods in East Asian populations. **(B)** Comparison of TMB between different sequencing methods in European and American populations. **(C)** Comparison of TMB between different racial groups. **(D)** Comparison of TMB between different racial groups in lung cancer.

To study the effect of different sequencing platforms and methods on TMB calculation, data of 4126 tumor samples sequenced with different platforms and methods were obtained. As shown in **Figure 1A**, no significant difference in TMB calculation from different sequencing platforms, including Illumina and MGI, was found. TMB from panel sequencing was higher than whole-exome sequencing (WES), however, there was no significant difference. To further verify the effect of different sequencing methods on TMB calculation, public sequencing data from 3680 samples performed with different methods were analyzed (**Figure 1B**). TMB values calculated from whole-genome sequencing (WGS) were significantly lower than those calculated from WES and panel sequencing. Furthermore, TMB in different races was analyzed. As shown in **Figure 1C**, TMB values of the East Asian populations were significantly lower than that of European and American populations in both WES sequencing and panel sequencing. The similar tendency was found in lung cancer (**Figure 1D**).

### TMB calculation was affected by the sequencing depth and tumor purity

**Figure 2:**
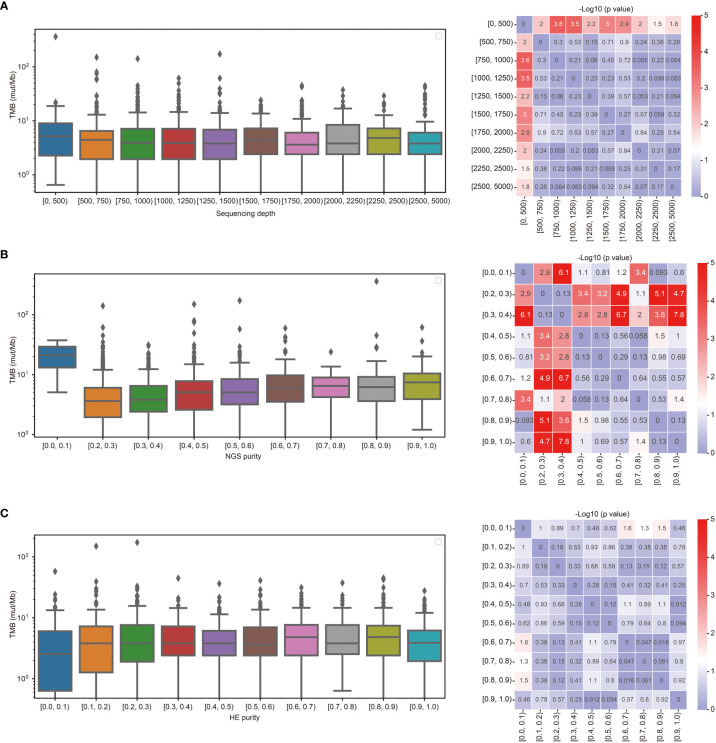
TMB calculation was affected by the sequencing depth and tumor purity. **(A)** Comparison of TMB between different sequencing depths. **(B)** Comparison of TMB between different purities estimated by NGS. **(C)** Comparison of TMB between different purities estimated by HE staining.

To investigate the effect of sequencing depth on TMB calculation, TMB calculated from lung cancer at different panel sequencing depths were analyzed. As shown in **Figure 2A**, the TMB calculated from sequencing depths ≥ 500 was significantly lower than that calculated from sequencing depths< 500. To determine the effect of tumor purities on TMB calculation, the distribution of TMB values with different tumor purities was analyzed. As shown in **Figure 2B**, the TMB values were higher than others for NGS purity between 0.0-0.1. As the NGS purity increased, the TMB values also tended to increase. However, the values of TMB were more stable with tumor purity ≥ 0.4. Compared with NGS tumor purity, The TMB calculation was less affected by the HE purity. There was no significantly difference with HE purity between 0.1-1.0 (**Figure 2C**).

### High correlation of TMB calculated with the optimized SGZ algorithm and paired normal-tumor sequencing in East Asian populations

**Figure 3:**
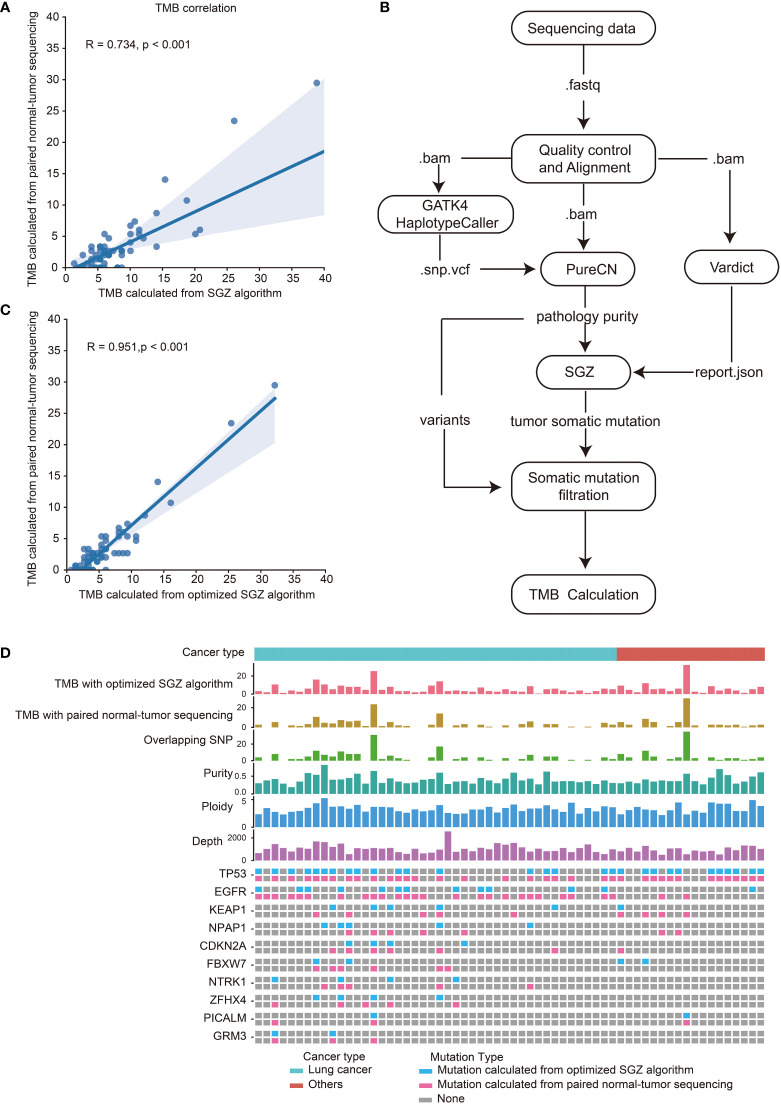
High correlation of TMB calculated with optimized SGZ algorithm and paired normal-tumor sequencing in East Asian populations. **(A)** Correlation of TMB calculated with the SGZ algorithm and the method of paired normal-tumor sequencing. **(B)** The process of TMB calculation from tumor-only sequencing samples with the optimized SGZ algorithm. **(C)** Correlation of TMB calculated with optimized SGZ algorithm and the method of paired normal-tumor sequencing. **(D)** The mutation landscape of sixty-two patients calculated with methods of optimized SGZ algorithm and paired normal-tumor sequencing. The top three histograms are the values of TMB calculated with the optimized SGZ algorithm, TMB calculated with the method of paired normal-tumor sequencing and overlapping SNPs calculated by both methods. Center three histograms are purity, ploidy and sequencing depth of samples. The mutation spectrum of each patient is shown under the value of sequencing depth. The upper row is the mutation detected by the method of paired normal-tumor sequencing, and the lower row is the mutation detected by the method of the optimized SGZ algorithm.

TMB calculation requires paired normal samples to remove germline mutations, which increases the cost of sequencing. In addition, in terms of clinical accessibility, paired normal samples are sometimes unavailable, which limits the clinical application of TMB. The SGZ algorithm for TMB calculation with tumor-only sequencing samples was designed based on European and American populations. To evaluate the accuracy of the SGZ algorithm for TMB calculation in East Asian populations, tumor tissues and matched peripheral blood samples from 62 patients including 43 lung cancer were collected and performed with targeted panel sequencing. The mean depths of tumor tissues and matched peripheral blood samples were 1027× and 455×, respectively (**Table S2**). As shown in **Figure 3A**, the TMB calculated by the SGZ algorithm had a low correlation with the TMB calculated by the method of paired normal-tumor sequencing in the East Asian populations. In order to calculate TMB from tumor-only sequencing samples in Asian populations, the SGZ algorithm was optimized. As shown in **Figure 3B**, we added the mutation filtering step, and constructed a background library with normal samples from East Asian patients to filter germline mutations, which ultimately reduced the false positives of TMB. To verify the accuracy of TMB calculation with an optimized algorithm, TMB calculated from the methods of the optimized algorithm and paired normal-tumor sequencing in sixty-two samples were compared. As shown in **Figures 3C**, **D**, their correlation coefficient is 0.95 and 82.7% of the mutations identified from the method of paired normal-tumor sequencing could be identified with the optimized SZG algorithm. These results demonstrate the accuracy of TMB calculation from the tumor-only sequencing with the optimized SGZ algorithm in East Asian populations.

### Identification of the TMB cut-off for predicting immunotherapy efficacy in the training cohort

**Figure 4:**
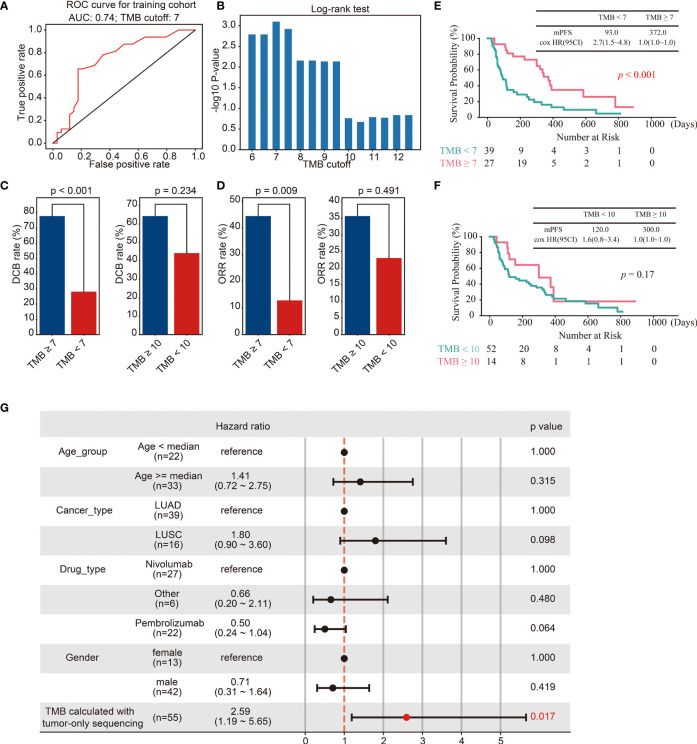
Identification of the cut-off of TMB for predicting immunotherapy efficacy in the training cohort. **(A, B)** ROC curves **(A)** and Log-rank test **(B)** for the identification of the TMB cut-off. **(C, D)** Barplots of DCB rate **(C)** and ORR rate **(D)** between different groups of TMB cut off 7 and 10. **(E)** Kaplan–Meier curves of OS comparing TMB ≥ 7 group and TMB< 7 group. **(F)** Kaplan–Meier curves of OS comparing TMB ≥ 10 group and TMB< 10 group. **(G)** The multivariate Cox regression analyses of the TMB, gender, age, drug, and cancer type.

The cut-off of TMB for predicting the efficacy of immunotherapy in European and American populations is 10 mut/Mb (19). However, since the TMB of East Asian populations is lower than that of European and American populations, a TMB cut-off of 10 mut/Mb may not be suitable for East Asian populations. To determine the cut-off of TMB for predicting immunotherapy efficacy in the East Asian population, tumor samples from sixty-six lung patients treated with ICIs were retrospectively collected as a training cohort. TMB was calculated with the optimized SGZ algorithm. The performance of TMB for predicting patient durable clinical benefit was analyzed with a ROC curve and Log-rank test. As shown in **Figure 4A**, the optimal cut-off of TMB was 7 mut/Mb with AUC = 0.74, and validated with Log-rank analysis (**Figure 4B**). Furthermore, the response rate and survival period were higher in patients with TMB ≥ 7 mut/Mb than in those with TMB< 7 mut/Mb, and the TMB cut-off of 7 mut/Mb is better than the TMB cut-off of 10 mut/Mb in East Asian populations (**Figures 4C–F**). To further investigate the role of TMB in predicting the efficacy of immunotherapy, the effects of TMB, medication type, tumor type and age group on patient survival were analyzed through multi-factor cox-regression. It was found that TMB was a favorable factor for patient survival, while other factors had no significant effect (**Figure 4G**).

### Validation of TMB cut-off in the test cohort

**Figure 5:**
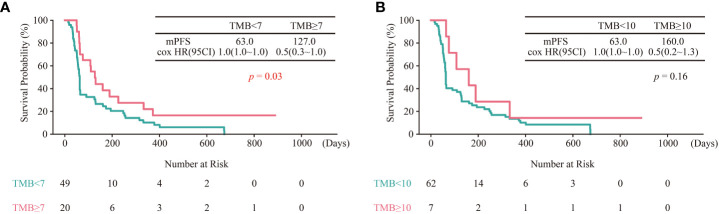
Validation of TMB cut-off of 7 mut/Mb in the test cohort. **(A)** Kaplan–Meier curves of OS comparing TMB ≥ 7 group and TMB< 7 group. **(B)** Kaplan–Meier curves of OS comparing TMB ≥ 10 group and TMB< 10 group.

To further validate the TMB cut-off of 7 mut/Mb, genomic and clinical data of sixty-nine East Asian NSCLC patients treated with ICIs were collected (29). Consistent with the above results, the survival period of patients with TMB ≥ 7 mut/Mb was longer than those with TMB< 7 mut/Mb (**Figure 5A**). Furthermore, the predicting efficacy with the TMB cut-off of mut/Mb is better than the TMB cut-off of 10 mut/Mb (**Figure 5B**).

## Discussion

Currently, different platforms and sequencing methods have been used to calculate TMB (21). However, the effect of different platforms and sequencing methods on the calculation of TMB is unclear. For the East Asian population, there are no methods to accurately calculate TMB from tumor-only sequencing samples and the optimal cut-off of TMB for predicting response to ICIs treatment is lacking. In this study, we have demonstrated that TMB calculation was not affected by different platforms, but was affected by different sequencing methods. Calculated TMB were more accurate and stable with sequencing depths ≥ 500, NGS purity ≥ 0.4 or HE purity between 0.1-1.0. After optimizing the SGZ algorithm, the correlation coefficient between TMB calculated from tumor-only sequencing samples and paired sequencing samples is 0.95. Through ROC curve and Log-rank test analysis, the cut-off for TMB was determined to be 7 mut/Mb in the training cohort. The TMB cut-off of 7 mut/Mb can better distinguish responders from non-responders than the TMB cut-off of 10 mut/Mb. Patients with TMB ≥ 7 mut/Mb experienced a higher response rate and survival period than those with TMB< 7 mut/Mb. Furthermore, genomic and clinical data of sixty-nine East Asian NSCLC patients treated with ICIs were applied to validate the TMB cut-off of 7 mut/Mb, and the same results were obtained.

Multiple studies have demonstrated that TMB is a predictive biomarker for immunotherapy in several types of cancers (15–17). However, consensus on how to measure TMB has not been reached. WES was considered the gold standard for TMB calculation. Compared with WES, panel sequencing has a shorter turnaround time and lower cost, thus increasing its clinical accessibility. However, whether the TMB calculated from panel sequencing could represent the TMB calculated from WES was unclear. Previous studies have shown a high concordance rate (R2 = 0.887) between TMB calculated from panel sequencing and WES, however, the samples measured were limited (35). In our study, no significant difference between TMB calculated from panel sequencing and WES was found.

When sequencing depth increases, mutations with low variant allele frequency (VAF) will be identified, which suggests that sequencing depth may have an impact on TMB calculation. A previous study has reported that multiple mutations were missed when the sequencing depth was between 100× to 200× (35). In our study, TMB calculated from WGS was significantly lower than those calculated from panel sequencing and WES, suggesting that the value of TMB would be affected by sequencing depth. Therefore, the effect of different sequencing depths on TMB calculation was systematically analyzed. It was found the calculated TMB was more stable with sequencing depths ≥ 500.

Due to the costs of sequencing and lack of matched normal samples, many clinical samples are tumor-only sequenced. At present, several algorithms were developed to calculate TMB from tumor-only sequencing samples (22–24). However, these algorithms were developed based on European and American populations, and are not suitable for East Asian people. In this study, the SGZ algorithm was optimized and a high concordance rate between TMB calculated from the methods of optimized SGZ algorithm and paired normal-tumor sequencing was found.

A previous study has investigated the racial differences in TMB and found that TMB cutoffs less than 10 mut/Mb may be more suitable for predicting response to ICIs in Asian populations (20). However, the optimal cut-off of TMB for predicting the efficacy of ICIs in the East Asian population is currently unclear. In this study, the TMB cut-off of 7 mut/Mb was identified in the East Asian population through the ROC curve and Log-rank analysis, which is less than 10 mut/Mb. Furthermore, this cut-off value was validated in another independent cohort.

There were several limitations in the study. First, the sample size used to correlate TMB calculated from tumor-only sequencing and paired sequencing was not very large, and further studies are needed to validate our optimized SGZ algorithm. Second, due to there was no other cohort that contained sufficient genomic and clinical data for patients with lung cancer in East Asian populations receiving ICIs, more researches are needed to further validate the cut-off value of TMB.

In summary, we have systematically evaluated the effect of different sequencing platforms and methods on the calculation of TMB, and optimized the SGZ algorithm. Furthermore, the cut-off of TMB to predict the efficacy of ICIs in the East Asian population has been identified and validated in another independent cohort. Ongoing intense work is needed to further validate and optimize the cut-off of TMB in the East Asian population who are treated with ICIs.

## Data availability statement

The datasets presented in this study can be found in online repositories via the following link: https://ngdc.cncb.ac.cn/gsa-human/browse/HRA003220.

## Ethics statement

The study involving human participants was reviewed and approved by YuceBio Ethics Committee (2021-003-04). The patients/participants provided their written informed consent to participate in this study.

## Author contributions

Conception and design: DS and HL. Acquisition of Data: DS, MX, and CP. Analysis and interpretation of data: MX, CP, HT, PW, and DW. Wrote the manuscript: MX, CP, and HT. Revised the manuscript: DS and HL. All authors have read and agreed to the published version of the manuscript.

## Funding

This study was supported by Natural Science Foundation of Tianjin (NO.20JCYBJC01350). 

## Acknowledgments

We would like to thank all the subjects and family members who gave their consent to present the data in this study, as well as the investigators and researchers at all relevant hospitals and study sites.

## Conflict of interest

Authors CP, HT, PW, DW, and HL were employed by the company YuceBio Technology Co.

The remaining authors declare that the research was conducted in the absence of any commercial or financial relationships that could be construed as a potential conflict of interest.

## Publisher’s note

All claims expressed in this article are solely those of the authors and do not necessarily represent those of their affiliated organizations, or those of the publisher, the editors and the reviewers. Any product that may be evaluated in this article, or claim that may be made by its manufacturer, is not guaranteed or endorsed by the publisher.
